# A Breastfeed-Promoting Mobile App Intervention: Usability and Usefulness Study

**DOI:** 10.2196/mhealth.8337

**Published:** 2018-01-26

**Authors:** Chih-Jau Wang, Pimwadee Chaovalit, Suporn Pongnumkul

**Affiliations:** ^1^ National Electronics and Computer Technology Center Pathum Thani Thailand

**Keywords:** mobile health, breast feeding, mobile applications, health promotion, usability, usefulness

## Abstract

**Background:**

Breastfeeding is proven to have lasting health benefits for both mothers and infants; however, 6-month exclusive breastfeeding rate remains below 20% in Thailand. Although the number of research literature and commercial apps for breastfeeding women is significantly growing, they are country-specific and restricted to English-speaking users. There exists a major knowledge gap on how mobile health apps could support breastfeeding in Thailand. To address these gaps, MoomMae has been developed with the intention to support Thai women in breastfeeding outside of their homes and in keeping their feeding records.

**Objective:**

The aim of this study was to evaluate the usability and usefulness of MoomMae, a mobile phone app designed to support breastfeeding women.

**Methods:**

Our study was reviewed and approved by Thailand’s National Science and Technology Development Agency (NSTDA) ethics committee. A total of 21 breastfeeding women with at least one Android phone or tablet were recruited via convenience and snowball sampling. The study process for each participant was as follows: the participant was requested to attend a preuse interview and given the app to use for 4 weeks. Following this period, a postuse interview was conducted to examine the usability and usefulness of the app. Both sessions were held individually and audiorecorded for qualitative analysis.

**Results:**

The mean scores of usability and usefulness from the postuse survey were 4.33 (SD 0.87; range 1-5) and 4.60 (SD 0.74; range 2-5). Our qualitative analysis revealed a total of 137 feedbacks: 71 related to usability and 66 associated with usefulness. A further sentimental analysis showed that comments on usability were generally negative (59 negative, 11 positive, and 1 neutral), and comments on usefulness were relatively positive (56 positive, 9 negative, and 1 neutral). We discovered 26 unique design issues and proposed recommendations for future improvement.

**Conclusions:**

Our usability and usefulness assessment of MoomMae demonstrated that MoomMae has a great potential to be a useful self-management tool for breastfeeding mothers in Thailand. The qualitative analysis suggested that the app is supportive of breastfeeding on demand, but the flow and inputs of the app should be redesigned to be more intuitive. For future implementations, the most desirable feature is a pump-reminding notification system.

## Introduction

### Breastfeeding

Breast milk is universally recognized as the best food for newborns. Studies have scientifically shown that breastfeeding provides optimal nutrients for infants, strengthens their immune system, and improves mother-and-child bonding [1,2]. Breastfeeding women also have lower risks of breast and ovarian cancer [3]. The World Health Organization (WHO), therefore, recommends exclusive breastfeeding for up to 6 months and continued breastfeeding for 1 to 2 years [4]. Despite the documented benefits, committing to breastfeeding can be challenging for mothers. As a result, exclusive breastfeeding rates at the recommended 6 months are under 39% at the global scale [5]. In Thailand, with an average of 715,000 newborns yearly between 2010 and 2015, the initiation rates of breastfeeding was 46% and the exclusive breastfeeding rates up to 6 months was only 12% [5].

One leading factor for discontinuation of breastfeeding is the discomfort to breastfeed or breast pump in the public [6,7]. Breastfeeding women described that they are judged or stopped from breastfeeding in the public as societies expect them to do it at home [8]. At the same time, mothers are expected to leave home for work, do grocery shopping, and take their babies to hospitals. Mothers often end up breastfeeding or breast pumping in public toilets or in personal cars, and some choose to give formula milk instead because of inconvenience [9].

Following a baby’s feeding pattern is another cumbersome task for mothers, as the quantity, duration, and frequency of feeding varies widely between mother-baby pairs and over time [10]. Some infants may spend 5 min on each breast and others can take up to 30 min per breast. Keeping a feeding record, therefore, helps mothers to learn when their child typically gets hungry and to know if their milk supply is sufficient. Sufficient milk supply consequently helps to build up the mothers’ confidence in their ability to breastfeed [11]. The feeding log also helps doctors to reassure that breastfeeding is going well and that the baby’s weight gain is on standard [12]. For instance, feedings that last more than 40 min repeatedly might indicate a sign of low milk supply or a baby’s wrong sucking skills.

### Information Technology for Breastfeeding Women

Research has shown that technological interventions were more effective in promoting breastfeeding when compared with traditional face-to-face interventions [13]. These technologies may vary in forms from message prompts [14,15], multimedia files [16,17], computer programs [18,19], to online websites [20-24]. In addition to this, mobile apps have become a popular platform used to support breastfeeding [25].

In the market, there exists a number of apps for breastfeeding mothers. Mobile apps for tracking feeding and pumping logs are vastly available. For example, Breastfeeding Tracker Pumping allows mothers to track the time and duration of different types of feeding [26]. Other apps come with the features of recording the baby’s feeds, sleep sessions, diaper changes, and growth [27-31]. Mobile apps for finding public breastfeeding places include MomsPumpHere, Mam Lactation, LatchMe in the United States [32-34], FeedFinder in the United Kingdom [35], Baby Places in Europe [36], MamaMap in Switzerland [37], and Nursing Room SG in Singapore [38]. However, these apps are specific to areas and countries. To the best of our knowledge, there is no such app in Thailand. Information regarding public breastfeeding rooms in Thailand are indexed online [39] and is shared via blogging such as Little Panda’s Travel Diary and Khajochi Blog [40,41].

### Usability of Mobile Health App

The advances in mobile technologies have made mobile phone apps more effective to promote health behavior change [42,43]. The ubiquitous nature of mobile phones has also promised greater patient engagement. However, its usability is often challenged by the hardware limitations, including small screen and limited input capabilities [44]. Studies have shown that mobile health (mHealth) apps are not always effective, and there is room for improvement [45,46]. New platforms should ideally be assessed to ensure usability.

Usability evaluation is the key to enhance acceptability and can be examined by several direct and indirect methods [47]. Two most common direct testing methods are thinking aloud and performance measurement. The thinking-aloud method is used to gain insights into real feelings of the subjects by asking them to navigate through the system and ask them to voice their thoughts aloud. In the performance measurement method, subjects are asked to perform a set of predefined tasks while investigators make some quantifiable measurements—such as completion time for each task. The performance measurement method is suitable for analyzing the usability of two competing approaches or designs. Indirect testing methods include questionnaire and interview. These methods are useful to assess parameters that are difficult to measure objectively, such as satisfaction and frustration.

Besides the direct and indirect protocols, heuristic evaluation has been applied to enhance user experience by identifying usability problems of a user interface (UI) that violate the design principles. The original heuristics was developed by Jakob Nielsen in the 1990s [48] with 10 heuristics to evaluate UIs on desktop devices. This set of heuristics, although widely used today, faces challenges to evaluate other devices. The research community has been putting efforts to modify these heuristics to be more suitable for mobile computing. In 2006, a group of researchers reported a new set of heuristics for mobile apps [49], which has been used in several studies [44,50,51].

### Objectives

Although the number of research literature and commercial apps for breastfeeding women is vastly expanding, these research and apps are restricted to English-speaking users. Existing apps for finding public breastfeeding places are also specific to countries. As a result, there is a concerning lack of the knowledge on how mHealth apps could support breastfeeding in Thailand. We address these gaps by evaluating usability and usefulness of MoomMae, a mobile app that aims to support women in Thailand to breastfeed comfortably in the public and keep their feeding and pumping logs efficiently.

## Methods

### Recruitment

A convenience and snowball sample of 21 breastfeeding women was enrolled in this study. Participants were approached via recruitment posters through two channels: 12 participants were members of a Facebook group named *Nom Mae Happy* (meaning happy breast milk) and 9 participants worked at National Science and Technology Development Agency (NSTDA), Thailand. The posters included the app introduction, inclusion criteria, and registration link. After registration, participants were contacted by telephone to give a brief introduction to the study and make an appointment for the subsequent interviews upon agreement with the subjects. Our inclusion criteria were as follows: subjects must be over 20 years old, own an Android mobile phone or tablet, live in Bangkok area, and be able to attend two 1-hour interview sessions. Participants were excluded if they were pregnant or were not competent to provide feedback by themselves.

### Ethical Review

This study was approved by the NSTDA ethics board of committee. The contents of participant information, informed consent form, recruiting posters, and registration form were reviewed. All participants provided full written informed consent before the interview sessions.

### Measurement and Analysis

**Figure 1:**
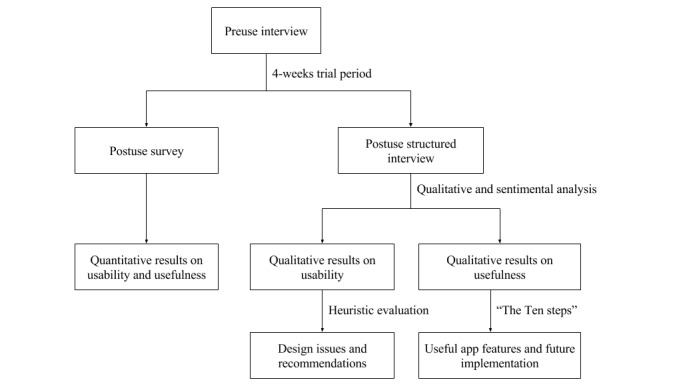
Evaluation flowchart.

Figure 1 summarizes the evaluation flow of our preuse and postuse structured interviews. Both sessions were audio-recorded, held individually, and led by the author who was not involved in the design and development of the app. The preuse sessions were held at the beginning of the 4-week trial period. On the day, a structured interview was performed to gather participant demographics, their current feeding options, prior experience with mobile phones, and expectation toward the app. After the 4-week app usage, the postuse sessions were conducted and consisted of two parts: a survey and a structured interview.

The postuse survey asked participants to rate usability and usefulness of the app based on the Likert scale. Possible scores were 1, 2, 3, 4, and 5, ranging from strongly disagree to strongly agree. Questions were adapted from a previously published study [52]. The usability questions compose of five attributes: learnability, efficiency, memorability, errors, and satisfaction. The usefulness questions were based on three criteria: app features, comfort after use, and intention to use. Results were analyzed to report means and standard deviations (SDs) of each component.

The postuse interviews were structurally performed and asked participants to share their insight into the app usage. Data collected through hand-written notes and recorded spoken responses were qualitatively analyzed. Each response was mapped to one app feature and labeled as positive, neutral, or negative. Results on usability were further analyzed using heuristic evaluation to identify design issues and to provide design recommendation. Results on usefulness were matched to the “Ten Steps to Successful Breastfeeding”, hereafter referred to as “The Ten Steps” [53], to reveal our roles in supporting breastfeeding and suggestions for future implementation.

### App Description

MoomMae is an mHealth app designed to serve as a supportive health technology for mothers to achieve their breastfeeding goals. The app consists of three main functions related to breastfeeding: feeding record, pumping record, and feeding rooms. In this study, we aim to evaluate the usability and usefulness of these three functions. Screenshots of the three functions in original language are available in Multimedia Appendices 1- 3.

**Figure 2:**
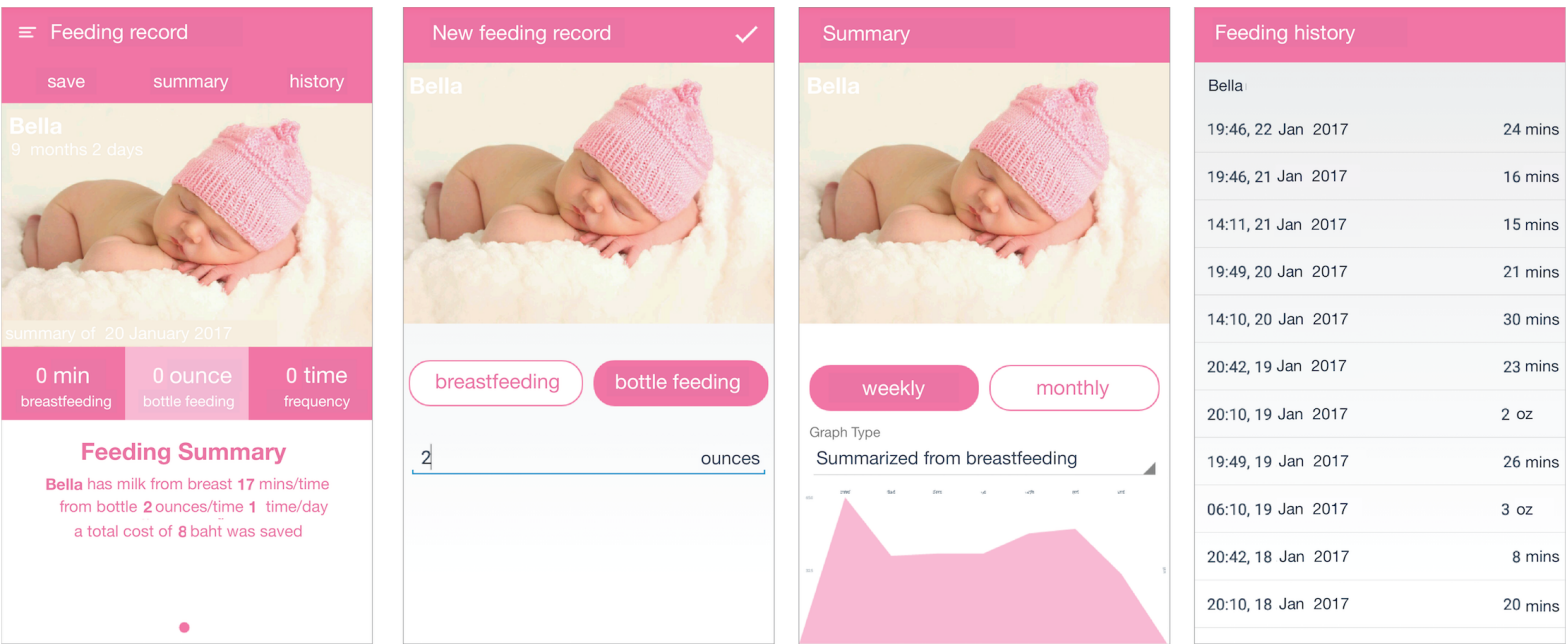
Screenshots of the “feeding record” function. (1) The main screen of the “feeding record” function; (2) Entering a new feeding log; (3) Summary of feeding record; (4) Feeding history. These screenshots are translated into English.

In the “feeding record” function (Figure 2; please refer to Multimedia Appendix 1 for the original language), mothers are able to insert new feeding records and view the summary or history of their logs. The main screen shows the daily and overall statistics of the records. Users can swipe left or right to go to the previous or the next child. Mothers can click the “save” button at the top of the screen to make a new feeding data. The “summary” button navigates to the summary page, which shows the graph of the feedings in weekly or monthly mode and in breastfeeding or bottle-feeding mode. The “history” tab leads to the history page, which lists all feeding data.

**Figure 3:**
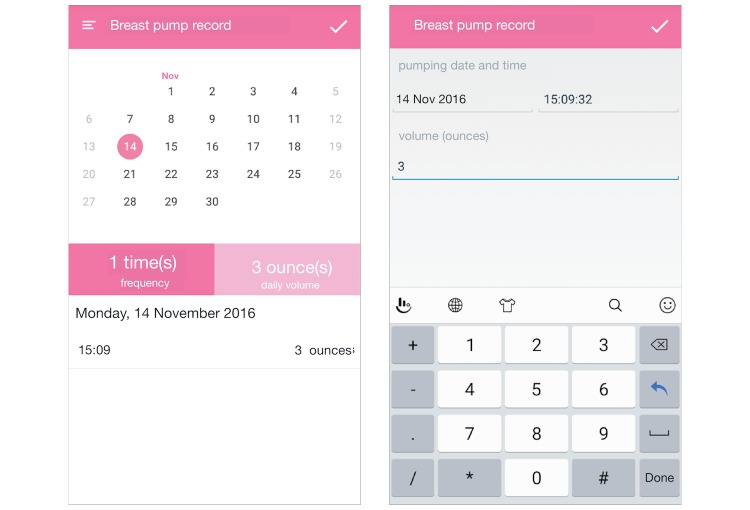
Screenshots of the breast pump function. (1) The breast pump function; (2) Making a new pumping record.

The “pumping record” function (Figure 3; screenshots in original language are available in Multimedia Appendix 2) allows mothers to insert pumping records and see daily statistics of their pumping records. The calendar at the top of the main screen is there for mothers to select a date to view their records or insert a new record. Once a date on the calendar is clicked, the daily statistics and list table view change to display only data of the selected date. To insert a new record, mothers can select a date on the calendar and click the check button at the top-right corner to go to the insert page.

**Figure 4:**
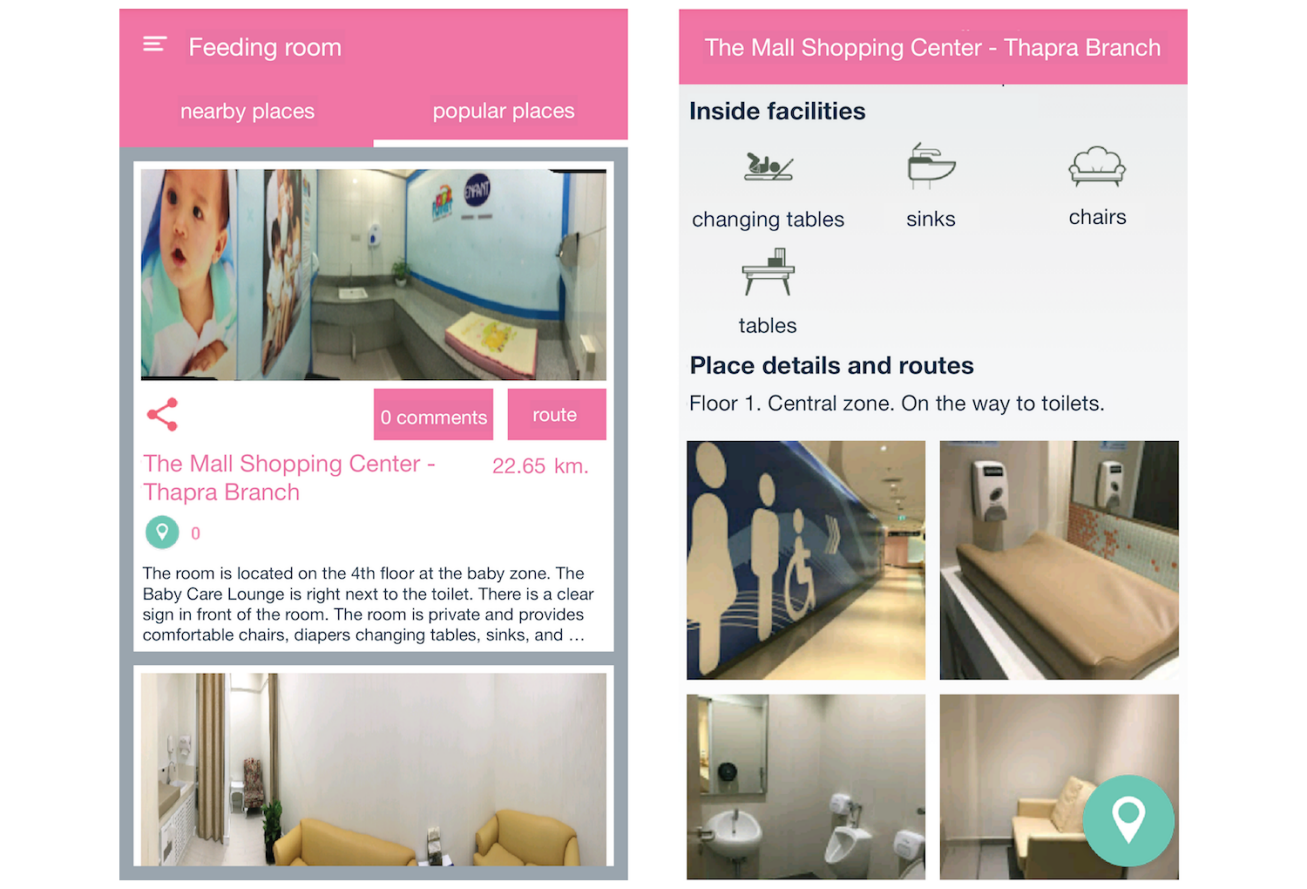
Screenshots of the breastfeeding rooms function. (1) The breastfeeding rooms function. (2) The detail page of a breastfeeding room.

The “feeding rooms” function (Figure 4; screenshots in original language are available in Multimedia Appendix 3) allows mothers to look for public feeding places. Mothers can click on the tabs at the top to change between nearby or popular feeding rooms. Once mothers click on a place on the list, the app will navigate to the detail page of that place that shows descriptions, facilities, routes, and photos of that room.

## Results

### Participant Characteristics

Table 1 summarizes demographic characteristics of 21 participants included in this study. The average age of the participants was 32.67 years (range 23-41). Almost all participants (20/21, 95%) exclusively breastfed up to 6 months. The remainder reported to breastfeed 98% of the time: giving formula milk a few times when her child was underweight. The leading motivation to breastfeed was to improve immune systems of their babies (20/21; 95%). The top three places to breastfeed were in personal cars (17/21; 81%), sitting areas in public places (13/21; 62%), and restaurants (13/21; 62%). Seven participants (33%, 7/21) used to note their feeding on papers, with one participant (s3) pointing out that it was difficult to calculate the sum and average on a notebook. Ten participants (48%, 10/21) had searched for a breastfeeding room by asking the staff of the building.

#### App Usage

In the preuse survey, 5 participants reported to spend 1 to 3 hours a day on mobile phones, 9 spent 3 to 5 hours, and 7 spent more than 5 hours. Participants were also asked to rate how much the following information would assist them to achieve their breastfeeding goals. On the scale of 1 to 5, the information about breastfeeding rooms, feeding records, and money saved received mean scores of 4.67 (SD 0.58; range 3-5), 4.38 (SD 0.92; range 2-5), and 4.62 (SD 0.80; range 2-5), respectively.

In the postuse survey, 16 participants (76%, 16/21) stated that they used the app 4 to 7 days a week. The other 5 participants (24%, 5/21) used the app 1 to 3 days a week. Only 4 participants immediately made a record in the app after they breastfed or breast pumped. Nine participants reported using the app during the night after their children fell asleep, stating it was the time they were more available. Six participants used the app in the morning, and 2 used the app during the daytime. The most frequently used functions for each participant were breastfeeding record (12/21, 57%), breast pump record (8/21, 38%), and breastfeeding room (1/21, 5%). The most satisfied functions for each participant were breastfeeding record (7/21, 33%), breast pump record (7/21, 33%), breastfeeding rooms (4/21, 19%), and breastfeeding summary (3/21, 14%).

### Quantitative Results

#### Usability

Usability scores and the complete question list used in postuse surveys are presented in Table 2. The total average score was 34.83 out of the full score of 40. Mean scores of each question were at least 3.60, which was above the midpoint of the scale 1 to 5. Not every question was answered by all participants. For instance, only a few participants used the app to look for public breastfeeding room; hence, Q4 and Q6 were answered by only 12 participants. The mean score for learnability, memorability, errors, efficiency, and satisfaction were at least 3.60 (SD 0.94), 4.76 (SD 0.54), 4.55 (SD 0.69), 4.48 (SD 0.87), and 4.33 (SD 0.73), respectively.

**Table 1:** Characteristics of the study participants.

Characteristic		Value
Age in years, mean (SD; range)		32.67 (4.50; 23-41)
**Education, n (%)**		
	≤High school diploma	2 (9)
	Bachelor’s degree	10 (48)
	Graduate degree	9 (43)
**Employment, n (%)**		
	Government officer	10 (47)
	Housewife	8 (38)
	Employee	1 (5)
	Self-employed	1 (5)
	Student	1 (5)
**Exclusive breastfeeding rates up to 6 months, n (%)**		
	100	20 (95)
	60-99	1 (5)
**Hours of mobile phone use per day, n (%)**		
	1-3 hours	5 (24)
	3-5 hours	9 (43)
	>5 hours	7 (33)

**Table 2:** Quantitative results of the usability survey.

Question	Usability attribute	n^a^	Mean (SD)^b^
1. You were able to learn and understand the app quickly when you first used it	Learnability	21	3.81 (1.03)
2. You think the other mothers can learn and understand the app quickly.	Learnability	20	3.60 (0.94)
3. If you were to have a period of not using the app, you think that you can easily pick it up again and remember how to use it	Memorability	21	4.76 (0.54)
4. The app accurately shows the information of public breastfeeding rooms	Errors	11	4.55 (0.69)
5. The app accurately shows your feeding history	Errors	20	4.80 (0.41)
6. The app helps you look for breastfeeding rooms faster	Efficiency	12	4.50 (0.80)
7. The app helps you to record your breastfeeding activities faster and more efficiently	Efficiency	21	4.48 (0.87)
8. Overall, you are satisfied with the app	Satisfaction	21	4.33 (0.73)

^a^Number of participants providing a response for each item.

^b^Out of 5. Responses for each item on a scale of 1 (strongly disagree) to 5 (strongly agree). Some participants indicated items were “not applicable” to their experience.

#### Usefulness

The results for usefulness (Table 3) were slightly higher than the usability scores in all aspects. The total average score was 36.81. The minimum mean score was 4.33, and the maximum mean score of 4.81. The mean score for app features, comfort after use, and intention to use were at least 4.38 (SD 0.81), 4.33 (SD 1.02), and 4.62 (SD 0.80), respectively.

### Qualitative Results

#### Design Issues

The postuse interviews exposed a total of 71 feedbacks on usability. A further sentimental analysis was conducted, and each comment was labeled as positive, neutral, or negative. The results showed that the majority of comments were negative—59 negative, 11 positive, and 1 neutral. Of the negative reports obtained, we revealed 26 unique design issues of the app that could affect the efficiency of use. The problems were then categorized by app features and mapped to one of the eight usability heuristics for mobile devices (Table 4).

The majority of the issues (8 out of 26) were identified to violate the second heuristics (H2)—match between system and the real world. The second most violated heuristics was H5 (6 issues), describing ease of input, screen readability, and glanceability and H6 (6 issues), describing flexibility, efficiency of use, and personalization. Following that, two issues were related to H8—realistic error management. The remaining four issues were associated with H1—visibility of system status and losability or findability of the mobile device, H3—consistency and mapping, H4—good ergonomics and minimalist design, and H7—aesthetic, privacy, and social conventions.

The frequency of these problems ranged from 1 to 6. The most occurring issue (frequency=6) was related to the process of making a new feeding record. The second most common issue (frequency=5) was on the subject of buttons, which do not resemble their actions. The third most expressed concerns (frequency=4) were associated with inputs, breaking down into inputs for volume, remark, and date. Looking at these problems, it is apparent that the app needs to be more intuitive.

Feeding and pumping record. Breaking down into app features, there were three design issues that appeared commonly in both “feeding record” and “pumping record” functions. First, 4 participants struggled to enter volume in ounces in decimal points. They felt that it was user error and they were the only ones who encounter this problem. Second, 4 participants mentioned their desires for writing down textual notes beyond numerical data, such as food they had on that day. Third, 3 participants had concerns with entering multiple records at once. Three participants stated the following:

I couldn’t enter decimal points, so I had to enter 0.5 ounces less for this time and added that extra 0.5 ounces to the next record. But, that made the data not accurate.

s15 (participant number); age 31 years; child 9 months

Making records on notebooks was more convenient. I could actually write everything I wanted to say about this record, including what I ate on that day.

s4; age 39 years; child 4 years

It took too long to enter a record. As a mother, I had too many responsibilities. I got kids to look after and housework to do. Sometimes, I couldn’t make a record right after I fed my baby. I ended up with too many records at the end of the day, but the app didn’t support entering multiple records at a time.

s13; age 28 years; child 5 months

##### Feeding Record

For the “feeding record” function, the majority had troubles with knowing where to click to make new (n=6) and past (n=3) feeding records. Another issue related to this function was the absence of saving status after the record has been entered. On the main screen, participants were not able to differentiate between the following items: daily and overall statistics, clickable button, and unclickable plain text. In the summary page, participants experienced difficulties with reading the graph. In the history page, participants struggled with seeking for a particular log as all data were listed on one page without any groupings. Four participants stated the following:

When I first entered the main screen of the feeding function, I didn’t know where to click to make a new record. The buttons at the top weren’t obvious enough.

s14; age 35 years; child 5 months

The app doesn’t allow me to enter a record for the past dates, so my data was incomplete.

s18; age 29 years; child 10 months

After I hit the save button, the app didn’t state if it was successfully saved or not. The app navigated back to the main screen, but still, I wasn’t sure if my records have been made or not.

s6; age 32 years; child 10 months

The feeding history page was really long and difficult to look for a record with a specific date. This made it difficult to change to date and time of the record when I needed to.

s13; age 28 years; child 5 months

**Table 3:** Quantitative results of the usefulness survey.

Question		n^a^	Mean (SD)^b^
**You think that the information in #1 to #4 helps you achieve in breastfeeding more:**			
	1. Information from searching for public breastfeeding rooms	14	4.57 (0.94)
	2. Information from recording amounts of both pumped and fed breast milk	17	4.59 (0.62)
	3. Information from recording duration of breastfeeding	16	4.38 (0.81)
	4. Information about cost you save from breastfeeding	8	4.75 (0.46)
5. You were more comfortable to go out		21	4.33 (1.02)
6. You were more comfortable to breast pump or breastfeed in public areas		21	4.76 (0.44)
7. You plan to continue to use the app		21	4.62 (0.80)
8. You plan to introduce the app to other mothers		21	4.81 (0.51)

^a^Number of participants providing a response for each item.

^b^Out of 5. Responses for each item on a scale of 1 (strongly disagree) to 5 (strongly agree). Some participants indicated items were “not applicable” to their experience.

**Table 4:** Usability issues resulted from interviews.

App feature and problem		Frequency	Heuristics^a^
**Feeding and pumping record**			
	Inputs for volume (in ounces) do not allow decimal points	4	H2—Match between system and the real world
	No free-text remark for feeding and pumping logs	4	H6—Flexibility, efficiency of use, and personalization
	Making multiple records at once is not possible	3	H6—Flexibility, efficiency of use, and personalization
**Feeding record**			
	The process of making a new feeding record is not intuitive	6	H2—Match between system and the real world
	The process of making past records is not intuitive	3	H6—Flexibility, efficiency of use, and personalization
	The graph in summary page is not informative	2	H2—Match between system and the real world
	No status shown after saving a record	2	H1—Visibility of system status and losability or findability of the mobile device
	The history page is difficult to read	2	H5—Ease of input, screen readability, and glanceability
	Daily and overall statistics shown on the main screen are difficult to differentiate	1	H5—Ease of input, screen readability and glanceability
**Pumping record**			
	The process of making a new pumping record is not intuitive	2	H2—Match between system and the real world
	Records are not listed in chronological order	1	H2—Match between system and the real world
**Feeding rooms**			
	Searching for a place by its name is not possible	2	H6—Flexibility, efficiency of use, and personalization
	Loading takes too long	2	H8—Realistic error management
	The lists of nearby and popular places are too informative and difficult to use	1	H4—Good ergonomics and minimalist design
**Other**			
	Inconsistent use of word choices referring to “breast milk”	2	H3—Consistency and mapping
	The buttons do not resemble their actions	5	H2—Match between system and the real world
	The registration process is not convenient	2	H2—Match between system and the real world
	Pressing the back button on the phone forces to exit the app	1	H2—Match between system and the real world
	When entering a name, hitting enter on keyboards goes to the next line instead of the next input	1	H2—Match between system and the real world
	The app forces to enter personal information in order to use other features	1	H6—Flexibility, efficiency of use, and personalization
	Date and time inputs are difficult to use	4	H5—Ease of input, screen readability, and glanceability
	Profile pictures are difficult to set up	2	H5—Ease of input, screen readability, and glanceability
	The menu icon is too small	1	H5—Ease of input, screen readability, and glanceability
	The Web page that the “breastfeeding information” function links to is not readable on mobile phones	1	H5—Ease of input, screen readability, and glanceability
	The app processes too slowly	3	H8—Realistic error management
	Some pictures are stretched to fit a fixed ratio	1	H7—Aesthetic, privacy, and social conventions

^a^Heuristics identified by a number preceded by an H (ie, H1: heuristic 1, H2: heuristic 2, etc.)

##### Pumping Record

The “pumping record” function consisted of two usability concerns. First, participants found the main screen difficult to navigate, and as a result, struggled to find the right place to click to insert a new pumping log. Second, records are sorted by the insert time instead of the pumping time. One participant stated the following:

When I first entered the pumping record function, I didn’t know where to click at all. I asked my husband to help but he couldn’t figure it out either.

s14; age 35 years; child 5 months

##### Feeding Rooms

In the “feeding rooms” function, participants experienced difficulties when they were going to a specific place and wanted to search in advance if there were any feeding rooms in that place. Others responded that the listing page took too long to load and was packed with too much information, making it difficult to read. Two participants stated the following:

When I needed to go to a particular place, I wanted to know if there is a feeding room there. However, there wasn’t any search boxes to search for a place by name. Thus, I needed to go back to Google.

s8; age 31 years; child 2 months

The feeding room function requires Internet. That makes the app not so effective when searching for a feeding room outside home. I would use my Wi-Fi at home to look for the place, and once I was there, I needed to ask the staff for more information.

s2; age 40 years; child 1 year 8 months

#### Design Recommendation

Our heuristic evaluation revealed the insights into barriers that prevent participants from undergoing joyful experience. A further analysis was carried out to provide a set of design recommendations that could be applied to similar systems and future versions of the app. The results are categorized by heuristics and presented in Table 5.

To improve user engagement, we recommend improving the flexibility of the following features: feeding record, feeding room, start-up page, and registration. For feeding record, the app should allow users to edit time when entering data, as most participants stated that they could not record right after feeding. We also suggest adding an optional text field for remarks of both feeding and pumping records. Furthermore, developers should implement a status bar indicating the status of saving or navigate the app to the location of the inserted record. For feeding room, we agree with participants that, to enhance efficiency, a search box should be added. In start-up page, designers should avoid forcing users to enter personal information to use other features; otherwise, a skip button should be provided. In registration page, the “I agree to terms” checkbox should be placed before the “register” button.

Displaying a long list of information is challenging and should be given extra attention to. Participants experienced difficulties in reading long lists of feeding history and feeding room exhibited in the app. We recommend that feeding records in the history page should be partitioned into smaller groups, ideally by feeding date. For feeding room list, essential information (eg, title) of each room should be enlarged and insignificant information (eg, number of comments) could be faded out to improve readability. Developers should also avoid loading data at once to enhance better loading time. Other minor advices include listing records in chronological orders and labeling all axes on the graph.

All UI components—such as buttons, input fields, and images—used in the app should be carefully designed. For instance, participants had troubles interpreting the meaning of the icons on the buttons; therefore, we encourage designers to place text labels next to the icons or else use text labels only. Important buttons should also be eye-catching and noticeable at the first glance. For input fields, we promote using the day-month-year date input for birthdays instead of the calendar-style date input. Developers should also implement input fields to support real-life conventions such as enabling decimal points for inputs with ounces as unit and replacing the “return” button on the keyboards with “next” when several fields are required. For images, the app should not stretch them to fit the screen width but display them in their original ratios. We recommend to avoid placing text on images and to replace missing ones by default pictures.

#### Usefulness of System Features

A total of 66 reviews on usefulness were uncovered from the postuse interviews. The obtained comments were grouped into 56 positive and 9 negative comments. When classified by the app features, the majority (36/66) were associated with the “feeding record” and “pumping record” functions. The remaining comments were expressed toward the “feeding rooms” function (19/66) and overall usefulness of the app (11/66).

##### Feeding and Pumping Records

For the “feeding record” and “pumping record” functions, there were 36 feedbacks captured and were sentimentally classified into 33 positive and 3 negative comments. The negative reviews were related specifically to the graph shown in the summary page, indicating that a more appropriate data visualization technique could be used. From the positive reviews, we discovered three benefits that the app provides: confidence building, feeding-and-pumping volume control, and time management. Participants reported that the app reduced their burdens of both insufficient and excess milk supply. Keeping a regular feeding and pumping records helped them estimate their children’s milk and manage their own milk supply more effectively. Additionally, continuation of recording helped mothers develop a more accurate feeding schedule and, consequently, enhanced their personal time management. Examples of participants’ feedbacks associated with the three themes mentioned above are illustrated in the following quotes:

The part of the app that show how much I saved was really pleasing. I gave my child the best food possible with the lowest price. It made me proud as a mother.

s14; age 35 years; child 5 months

Keeping a feeding record helps me notice the feeding time. In the past, my mom fed my kid every time he cried. She thought he was hungry, but that wasn’t always the case. Now, I know his time. I won’t overfeed him and his weight won’t exceed the standard.

s14; age 35 years; child 5 months

My kid was sick, and my doctor kept asking if he ate less than he usually did. I didn’t think he could eat less. I thought he ate normally. Now that I start using the app, I can clearly see that his eating trend is dropping.

s13; age 28 years; child 5 months

**Table 5:** The design recommendations.

Heuristics^a^	Design recommendation
H1—Visibility of system status and losability or findability of the mobile device	Implement a status bar or navigate the app to the location of the inserted record
H2—Match between system and the real world	Make sure the add button is obvious and noticeable at the first glance List all records by chronological order Enable decimal points for ounces input fields Make sure icons on buttons resemble their actions. Include text on buttons if appropriate Specify the input method type to replace the return button on keyboards with done or next Label the graph axes
H3—Consistency and mapping	Use the word “breast milk” instead of “milk” alone. If appropriate to the context, apply it across the app
H4—Good ergonomics and minimalist design	For each item on the feeding rooms list, enlarge important information (such as title) and fade out insignificant information (such as the comment count)
H5—Ease of input, screen readability, and glanceability	In feeding history, group feeding records by dates and feeding method Avoid putting text on images In profile setup page, omit the calendar input. Use regular day/month/year date input Make sure important buttons are eye-catching and noticeable at the first glance
H6—Flexibility, efficiency of use, and personalization	Add a free text field for remarks of each feeding and pumping record Redesign the process of making new feeding records. Allow users to edit time while entering data Add a search box at the top of the feeding room list Avoid forcing users to enter personal information or provide a skip button Place the “I agree to terms” checkbox before the “register” button
H7—Aesthetic, privacy, and social conventions	Avoid stretching images to fit the screen size. Replace missing image with appropriate default image
H8—Realistic error management	In feeding rooms list, avoid loading all information at once

^a^Heuristics identified by a number preceded by an H (ie, H1: heuristic 1, H2: heuristic 2, etc.)

##### Feeding Room

A total of 14 positive and 5 negative comments were expressed toward the usefulness of the “feeding room” feature. The negative feedbacks are related to the limited number of feeding rooms exhibited in the app, especially to the area outside of downtown Bangkok. Of the positive comments, participants indicated that the information about feeding rooms made breastfeeding in the public more effective, pleasant, and comfortable, as illustrated in the following quotes:

Previously, I needed to carry a piece of cloth with me everywhere to cover my breast. Now, I can look or feeding places in advance. I can manage myself better. I knew where and when to feed my kids. I knew what tools and equipment to bring with me.

s20; age 33 years; child 3 months

The app made me less worried about breastfeeding in the public. Before I use the app, I always needed to hide in my car. I ended up going to the same mall again and again. It was troublesome to me and to my family members. Now that I use the app, I have more options. My house is really close to the Emporium shopping mall, but I have never known that there is a feeding room there. I don’t have to go to the same department store again and again.

s10; age 31 years; child 1 year

It brings mothers to a place that everyone is so supportive of breastfeeding. Being there, I didn’t have to care what people would think about breastfeeding a 4-year-old child.

s4; age 39 years; child 4 years

#### Intention to Use

After all, although there exist some design issues, these problems did not cause severe inconveniences in using the app. Both quantitative and qualitative results reflected that participants actually found the app acceptable and had a high intention to continue using the app after the trial period. Moreover, they also believed that the app would be beneficial to other mothers in general, as illustrated in the following quotes:

My life is easier after using the app, so I will definitely continue using this app.

s20; age 33; child 3 months

I will introduce this app to my friends. It really helps mothers recording data to show doctors.

s7; age 34; child 10 months

## Discussion

Breastfeeding women from Bangkok and the metropolitan region were recruited to qualitatively examine the usability and usefulness of MoomMae, a mobile phone app designed to promote breastfeeding in Thailand. To maximize the real-life experience, the app was installed on participants’ devices and was given to them to use in their daily life for 4 weeks. Individual structured interviews were conducted at the beginning and the end of the trial period. The quantitative results showed a high usability and usefulness score. The qualitative findings provided the insights into usability issues and usefulness of the app.

### MoomMae’s Role in Supporting Breastfeeding

The Ten Steps to Successful Breastfeeding were developed by WHO and Unicef as a guideline for health facilities to follow to support mothers in breastfeeding [53]. Four of the ten steps (#3, #5, #8, and #10) are not specific to breastfeeding in hospitals and should be continued after discharge. Our findings suggested that MoomMae strongly supports step 8 (encourage breastfeeding on demand) and partly supports step 10 (foster the establishment of breastfeeding support groups and refer mothers to them on discharge).

#### Self-Efficiency

Our analysis indicates that MoomMae addresses challenges mothers face while breastfeeding. Being a mother is difficult and stressful because of the numerous tasks needed to be achieved daily. MoomMae promotes self-efficiency by helping them to keep things in shape. The numerical values derived from feeding and pumping records help mother to learn their babies and helps manage their schedule. The ubiquitous nature of mobile phones also makes the app more efficient than the notebook approach, as illustrated in the following quote:

The app was more convenient. I used to record it on notebooks, and sometimes I was just too lazy to go get my notebook. Using the app is different since I always use my phone while pumping anyways. I can just make a record after I finish pumping.

s17; age 32 years; child 3 months

#### Locality and Public Awareness

The locality of MoomMae is the key to promote breastfeeding in Thailand. MoomMae is the first mobile app that index public breastfeeding places in the local context. We learned from the preuse interviews that it is the most desirable function, and mothers expected it to be the most useful feature. Although the postuse interviews showed that only 11 (out of 21) participants used the feature, participants did not complain about the way the app presented information. Rather, the feedback was regarding the lack of feeding rooms. Moreover, the Thai language used in the app is another key factor that makes MoomMae localized and unique to Thai users, as illustrated in the following quote:

The app is really easy to use. I don’t find any problems. And, everything is written in Thai, so it’s much easier. It’s unlike those foreign apps that are written in English.

s16; age 31 years; child 1 year 6 months

More importantly, our analysis suggested that MoomMae has the potential to raise public awareness toward breastfeeding. Participants reported that the existence of the app showed them that breastfeeding is so widespread that there is an app developed to support it. One participant described that as more mothers use the app to find feeding rooms, public places would give extra attention to building breastfeeding rooms, and consequently, the public will gradually view breastfeeding as a norm.

### Future Implementation

Postuse interviews and The Ten Steps analysis exposed shortcomings of MoomMae that could be improved in future versions. Participants raised the ideas of making the app send a notification to remind them about their pumping schedules, which supports step 5 in The Ten Steps: show mothers how to breastfeed and how to maintain lactation even if they should be separated from their infants. Subjects also want the app to provide question and answer chat rooms, which is associated with step 10: foster the establishment of breastfeeding support groups and refer mothers to them on discharge from the hospital or clinic. Another suggestion from participants is to include information about how to increase or decrease their milk supply, which matches step 5: inform all pregnant women about the benefits and management of breastfeeding. For UI modifications, participants suggested adding cartoon characters and using distinct colors to contrast different functions. From the authors’ points of view, our suggestion would be enhancing the existing strength of the app. For instance, as participants found calculating the sum of their feeding records on the app easier, the app can calculate these numbers and display it on the app automatically.

### Limitations

We acknowledge that it is difficult to generalize our findings because of small sample size and nonprobability sampling methods. However, prior usability studies have suggested that a small number of participants, as few as 5 to 20, with similar background show great potential for exploring a variety of perspectives that can identify a vast amount of design problems [54,55]. In addition, because of scheduling and transportation constraints of breastfeeding women, it is almost impossible to recruit probability samples. The convenience sampling is, therefore, more feasible and used in previous literature [56-58].

Our study is also limited by the participant characteristics, which may not reflect the whole population of Thai mothers. The exclusive breastfeeding rates of our samples are close to 100%, which is much higher than the rate (12%) of the majority of Thai mothers. We initially targeted to recruit mothers with varied breastfeeding intentions; however, we only received registrations from mothers with high intention in breastfeeding. This can be explained that mothers with already high intention to breastfeed responded to our recruit posters more quickly and were very interested and responsive in breastfeeding-related studies. Furthermore, our participants were slightly older than the average Thai mothers. In 2015, the mean age of mothers of newborns was 27.5 years [59]; however, our participants gave birth to their current children at the average age of 31.6 years.

We understand that the interview method may not be the ideal protocol for usability evaluation, but it was the most appropriate method after deliberate considerations. For focus group method, the research team thinks that breastfeeding mothers are always busy and have varied schedule to be involved in focus groups. The performance measurement method may not achievable as breastfeeding mothers may need to take care of their children while meeting the researchers, that is, some mothers bring along their children to the research sessions. We also considered that performing a thinking-aloud test in the field may be too noisy and distracting. Taken together, the structured interview was chosen to be the most appropriate method.

### Conclusions

Breast milk is universally known to be optimal food for infants. Despite this promise, persisting with breastfeeding is challenging, and exclusive breastfeeding rates remain low in Thailand. To the best of our knowledge, the existing research and commercial apps are country-specific, and there exists a major knowledge gap on how mHealth apps could support breastfeeding in Thailand. MoomMae has therefore been developed and evaluated to support mothers in breastfeeding in the public and in keeping their feeding records. We recruited 21 breastfeeding mothers to participate in preuse and postuse interviews to qualitatively evaluate usability and usefulness of the app.

This study contributes to the growing literature demonstrating how usability assessment of mHealth apps provides invaluable information for iterative developments. The results suggested that MoomMae is easy to use and has the potential to be a useful app for breastfeeding mothers in Thailand. One of the strengths of the app is to support breastfeeding on demand. However, making the flow and inputs more intuitive could enhance the user engagement. Our study also suggests several recommendations for future implementation. Future studies could attempt to recruit participants from a wider range of backgrounds, and other in-depth evaluation methods could be carried out.

## Appendix

Multimedia Appendix 1 Screenshots of the “feeding record” function (in Thai).

Multimedia Appendix 2 Screenshots of the “breast pump” function (in Thai).

Multimedia Appendix 3 Screenshots of the “feeding room” function (in Thai).

## Abbreviations

mHealth: mobile health
NSTDA: National Science and Technology Development Agency
SD: standard deviation
UI: user interface
WHO: World Health Organization
