# Supplementary figures and images for: A Breastfeed-Promoting Mobile App Intervention: Usability and Usefulness Study
Source: JMIR Mhealth Uhealth. 2018 Jan 26;6(1):e27. doi: 10.2196/mhealth.8337 (PMC5807626; doi:10.2196/mhealth.8337)

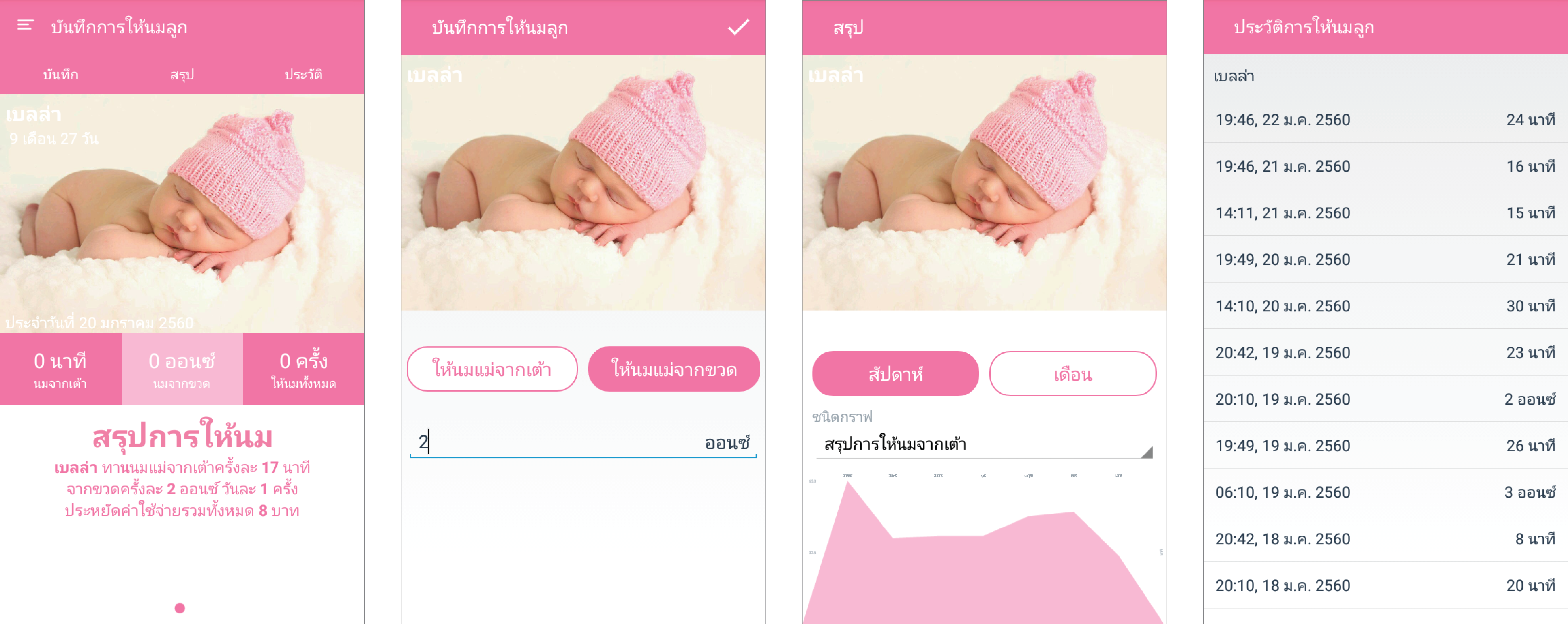

Supplement: Multimedia Appendix 1 [file mhealth_v6i1e27_app1.png]

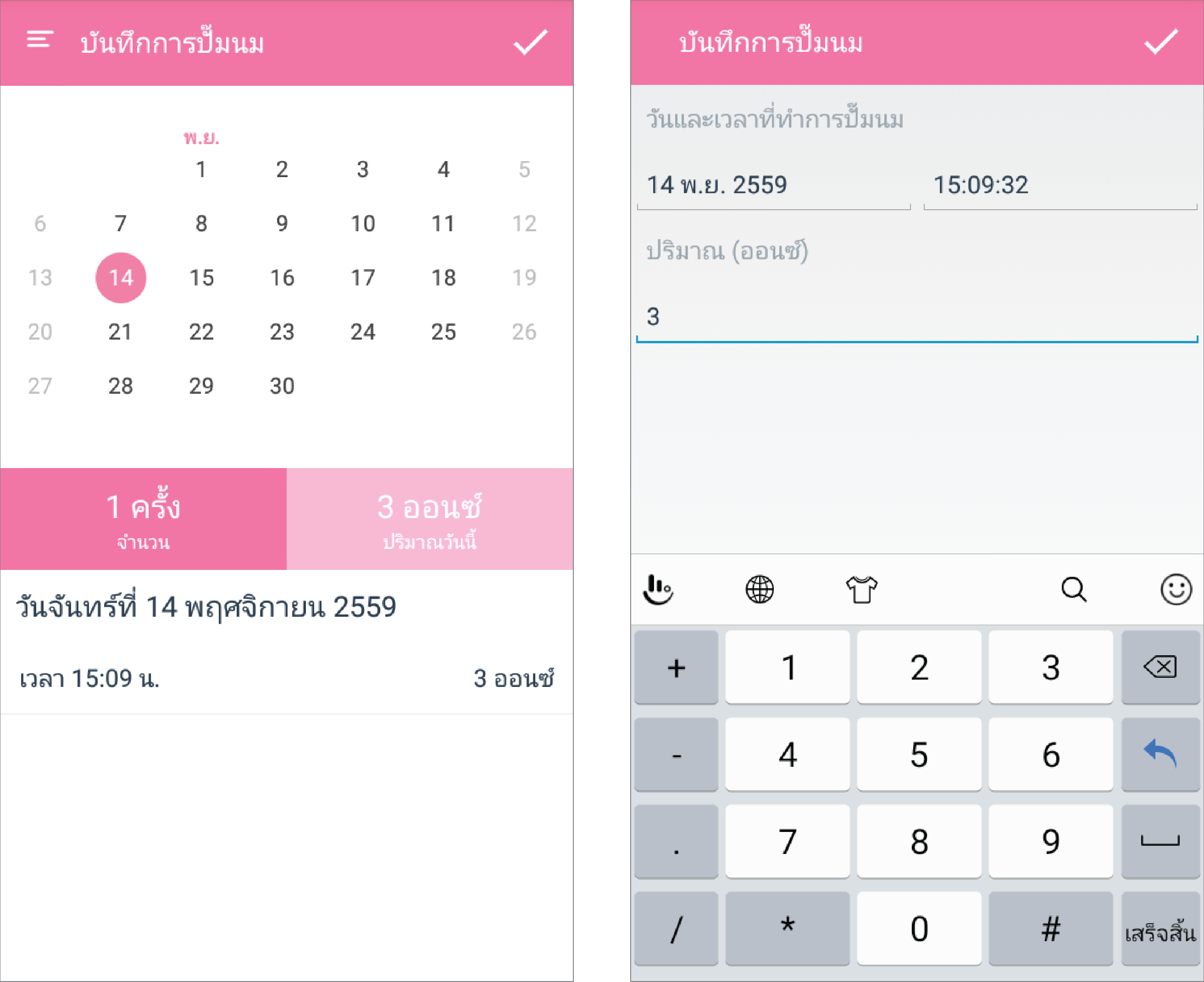

Supplement: Multimedia Appendix 2 [file mhealth_v6i1e27_app2.png]

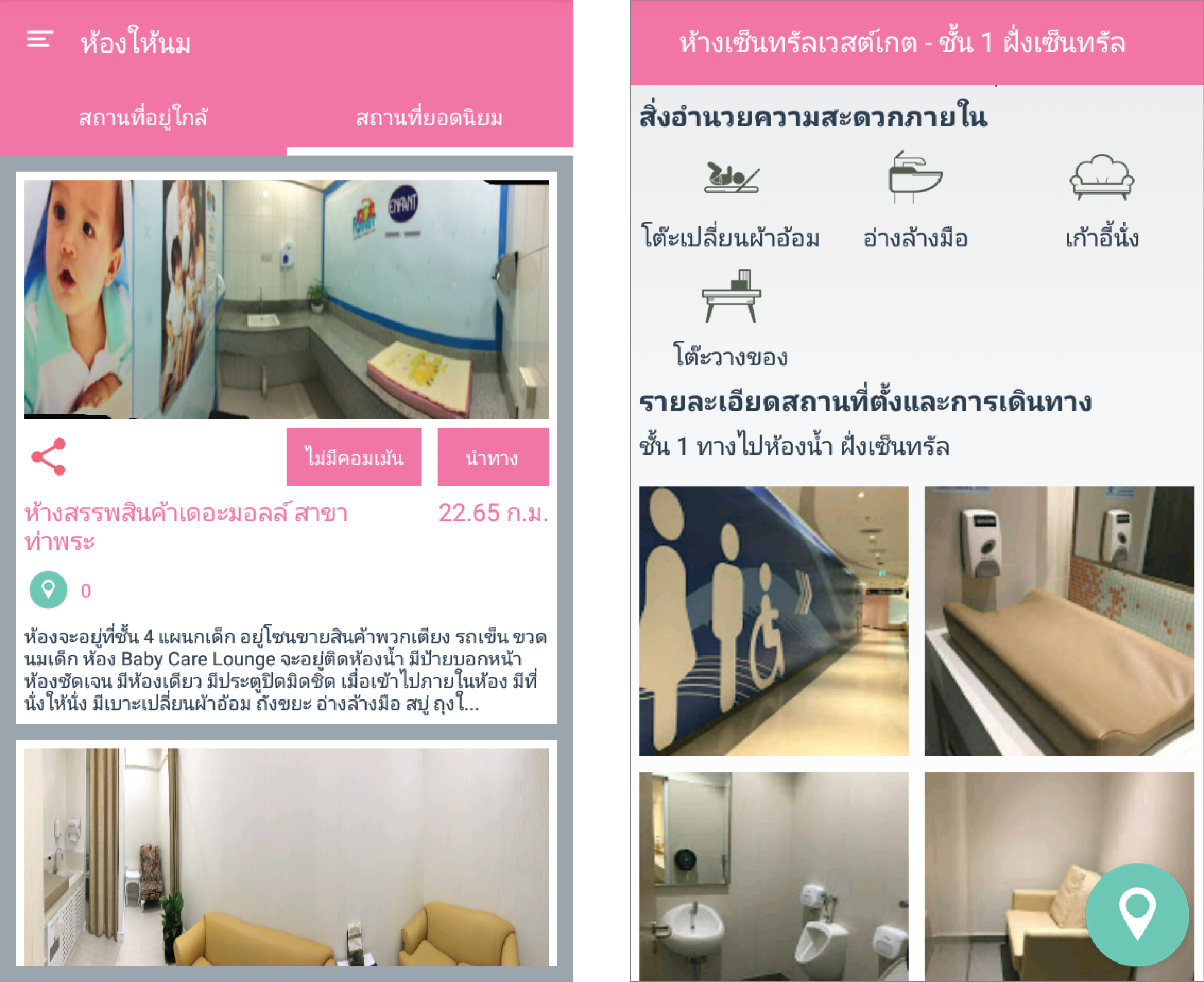

Supplement: Multimedia Appendix 3 [file mhealth_v6i1e27_app3.png]
